# The DAPA‐DIET study: Metabolic response to Dapagliflozin combined with dietary carbohydrate restriction in patients with Type 2 Diabetes Mellitus and Obesity—A longitudinal cohort study

**DOI:** 10.1002/edm2.381

**Published:** 2022-10-20

**Authors:** Petra Hanson, Harpal Randeva, Dan J. Cuthbertson, Paul J. O'Hare, Nick Parsons, Kamaljit Chatha, Gemma Reidy, Martin O. Weickert, Thomas M. Barber

**Affiliations:** ^1^ Warwick Medical School University of Warwick Coventry UK; ^2^ Warwickshire Institute for the Study of Diabetes Endocrinology and Metabolism University Hospitals Coventry and Warwickshire Coventry UK; ^3^ Institute of Cardiovascular and Metabolic Medicine University of Liverpool Liverpool UK; ^4^ Department of Endocrinology Liverpool University Hospital NHS Foundation Trust Liverpool UK; ^5^ Biochemistry and Immunology Department University Hospitals Coventry and Warwickshire Coventry UK

**Keywords:** appetite, carbohydrate restriction, energy expenditure, fat mass, lean mass, metabolism, SGLT2 inhibitor, type 2 diabetes

## Abstract

**Objective:**

The cardio‐renal benefits of sodium glucose‐like transporter 2 inhibitor (SGLT2i) therapies have been demonstrated in patients with and without type 2 diabetes. However, no studies have explored the long‐term metabolic effects of SGLT2i, combined with dietary carbohydrate restriction. Our primary objective was to describe long‐term changes in weight, energy expenditure, appetite and body composition after 12 months of Dapagliflozin therapy, with carbohydrate restriction, in people with type 2 diabetes and obesity. Our secondary objective was to assess changes in adiponectin and leptin.

**Method:**

This was a 12‐month cohort study in a secondary care setting. Participants (*n* = 18) with type 2 diabetes (T2D) and class 3 obesity underwent baseline indirect calorimetry for determination of 24‐h energy expenditure, body composition, fasting serum leptin and adiponectin levels, and appetitive assessments. Following initiation of Dapagliflozin (and dietary carbohydrate restriction), measurements were repeated at monthly intervals up to 12 months.

**Results:**

Mean starting weight of participants was 129.4 kg (SD 25.9), mean BMI 46.1 kg/m^2^ (SD 8.3) and mean HbA1c 53.9 mmol/mol (14.1). Seventeen participants completed the study; after 12 months of Dapagliflozin and dietary carbohydrate restriction, mean weight loss was 8.1 kg (SD 11.3 kg; *p* = .009). This was mediated by reduced fat mass (mean loss, 9.9 kg; SD 10.4 kg; *p* = .002) associated with reduced serum leptin at 12 months (mean reduction 11,254 pg/ml; SD 16,075; *p* = .011). There were no significant changes in self‐reported appetite, 24‐h energy expenditure or serum adiponectin during follow‐up.

**Conclusion:**

In this study, combined Dapagliflozin therapy and carbohydrate restriction in patients with T2D and obesity resulted in a significant reduction of body weight and fat mass at 12 months without any discernible changes in energy expenditure or appetite. These results offer a scientific and clinical rationale to conduct an exploratory trial investigating the effects of a low carbohydrate diet combined with SGLT2 inhibitors in patients with T2D.

## Graphical abstract

SGLT2i leads to a sustained weight loss among patients with type 2 diabetes (T2D), with no changes in energy expenditure in the first 12 months of therapy. Combination of the SGLT2i Dapagliflozin and carbohydrate restriction in patients with T2D and obesity resulted in a significant reduction of weight, fat mass and serum leptin, with no discernible change in energy expenditure or appetite at 12 months. Reduced carbohydrate diet with SGLT2i among patients with T2D and obesity could lead to a more clinically meaningful weight loss without detrimental effect on appetite and energy expenditure. These results offer a scientific and clinical rationale to conduct an exploratory trial investigating the effects of a low carbohydrate diet combined with SGLT2 inhibitors in patients with T2D.

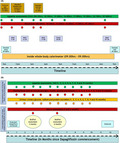

**Novelty statement**

**What Is Already Known?**

- SGLT2i therapies lead to weight loss among patients with type 2 diabetes (T2D).

**What this Study Has Found?**

- Combination of the SGLT2i Dapagliflozin and carbohydrate restriction in patients with T2D and obesity resulted in a significant reduction of body weight, fat mass and serum leptin, with no discernible change in energy expenditure or appetite at 12 months.

**What Are the Implications of the Study?**

- Reduced carbohydrate diet with SGLT2i therapy among patients with T2D and obesity could lead to more clinically meaningful weight loss without detrimental effects on appetite and energy expenditure. These results offer a scientific and clinical rationale to conduct an exploratory trial investigating the effects of a low carbohydrate diet combined with SGLT2inhibitors in patients with T2D.

## 1 INTRODUCTION

The Sodium‐Glucose‐like Transporter 2 Inhibitor (SGLT2i) class of drugs is an oral therapeutic option for patients with T2D.^1^ The glycaemic effects of SGLT2i therapies occur through the promotion of urinary glucose excretion.^2^ Recent data from large cardiovascular outcome trials highlight significantly improved cardiovascular and renal outcomes in patients with T2D treated with SGLT2i.^3^ Furthermore, SGLT2i facilitate secondary weight loss.^4^ Two proposed mechanisms for weight loss include (i) urinary excretion of calories from glycosuria^4^ and (ii) fluid loss (supported by an observed 7.3% reduction in plasma volume from 12 weeks of SGLT2i therapy).^5^ However, there is mixed evidence in the literature regarding the temporal pattern of weight loss with SGLT2i. Some studies suggest a plateauing effect at 24 weeks post‐initiation.^6^ Other studies show continual weight loss up to 102 weeks post‐initiation.^7^ Bailey et al.^8^ showed a ‘dual‐phase’ effect, with early rapid weight loss followed by a progressive reduction in body weight and decreased waist circumference in response to Dapagliflozin.

The mechanism(s) for the observed weight loss in response to SGLT2i therapies remains incompletely understood. Following therapeutic blockade of renal proximal tubule SGLT2 receptors, ~50% of renal glucose within the ultrafiltrate (approximately 80 g) is reabsorbed by SGLT1 receptors in the context of normoglycaemia. Accordingly, an observed urinary glucose loss of 80–120 g/day with SGLT2i in patients with T2D would translate into a daily caloric deficit of 320–480 kcal, and a predicted weight loss of between 5.5 and 7 kg in 3–4 months,^4^ exceeding the weight loss reported in the literature.^4^, ^6^, ^7^ Although the discrepancy between observed and expected weight loss could manifest from up‐regulation of SGLT1 receptors, it is feasible that adaptations to metabolic rate and appetite may attenuate SGLT2i‐induced weight loss.^4^

Given the paucity of long‐term studies reporting on the metabolic effects of SGLT2i, our aim was to characterize the longer‐term changes in metabolic rate, appetite and body composition with Dapagliflozin therapy combined with dietary carbohydrate restriction in participants with T2D and class 3 obesity.

## 2 METHODOLOGY

### 2.1 Ethics statement

Our longitudinal cohort study was approved by East Midlands Research Ethics Committee in September 2017 (IRAS 229929, REC 17/EM/0330). Local approval by the Research and Development department at University Hospitals Coventry and Warwickshire (UHCW) was obtained in October 2017, and the study was sponsored by the University of Warwick (SC.80/16‐17).

### 2.2 Recruitment

We recruited 18 adult patients (age 18–75 years) with established T2D and class 3 obesity (BMI ≥ 35 kg.m^−2^), who attended hospital‐based weight management out‐patient clinics at the Warwickshire Institute for the Study of Diabetes, Endocrinology and Metabolism (WISDEM), UHCW, between December 2018 and December 2019. All recruited participants were suitable for initiation of Dapagliflozin. All participants were naive to injectable therapies (including insulins and Glucagon‐Like Peptide 1 [GLP1] therapies), SGLT2i and Dipeptidyl Peptidase‐4 (DPP4) inhibitors. All inclusion and exclusion criteria are summarized in Table 1.

**TABLE 1:** Inclusion and exclusion criteria

Inclusion criteria	Exclusion criteria
Adult patient (age ≥ 18 years) with an established diagnosis of T2D	Any pre‐existing use of drugs within the SGLT2 inhibitor or DPP4‐inhibitor classes
Clinical need for initiation of Dapagliflozin based on inadequate glycaemic control	Children or female patients who are pregnant or breast‐feeding
Decisional capacity and ability to provide informed consent	Elderly patients >75 years (due to lack of clinical data on efficacy of therapies)
Naive to injectable therapies including insulin and GLP1 agents	Evidence of moderate to severe heart failure
Pre‐existing metformin and Sulphonylurea use is acceptable	Any concomitant use of loop diuretic therapies or pioglitazone (as per licensing for Dapagliflozin)
Obesity (BMI ≥35Kgm^−2^)	eGFR<60 ml/min/1.73 m^2^ (either at the time of initiation of Dapagliflozin or during the 12‐month treatment period with Dapagliflozin)
Adequate Renal function, eGFR>60 ml/min/1.73m^2^	History of severe or recurrent urinary‐ or genital‐tract infections
	History of bladder cancer

### 2.3 Research protocol

This was a longitudinal cohort study. 24‐h profiles of metabolic rate at baseline and at 12‐months (following initiation of Dapagliflozin) were performed on the Human Metabolism Research Unit (HMRU). Each participant had measurements of body weight, appetite and fasting blood tests at baseline and at monthly intervals until 10 months and then at 12 months. Participants underwent assessment of body composition at baseline and at months 3, 6 and 12.

### 2.4 Body composition assessment

‘Body composition tracking system’ (BOD POD® Life Measurement Inc, Concord, California, USA) was used for assessment of body composition. This uses whole‐body air‐displacement plethysmography, with whole‐body densitometry to determine body composition.^9^ BODPOD uses air instead of water to measure body volume (based on the principle of Boyle's Law) and is a reliable and valid technique for measuring body composition in individuals with obesity.^10^

### 2.5 Whole‐body indirect calorimetry

For assessments of 24‐h energy expenditure, we used whole‐body indirect calorimetry,^11^ located in HMRU. Measurements take place in an airtight respiratory chamber, which is part of an open circuit ventilated indirect calorimeter allowing constant monitoring of oxygen and carbon dioxide. The method was described by Ravussin et al.^12^ and involves using a thermomagnetic oxygen analyser (Magnos 2 T, full scale range 19–21%) and infrared carbon dioxide analyser (Uras 2 T, full scale range 0%–1%). Calculation of rates of change of oxygen and carbon dioxide concentrations enables accurate estimation of energy expenditure.

The airtight respiratory chamber within HMRU has a large window, 2 airtight compartments where exchange of food, containers and other items can occur. Communication was enabled via intercom, but if intercom was not in use the chamber was sound proof. An air conditioning system maintained the temperature within the chamber at 24°C throughout all studies. Blood samples were obtained through a small opening in the door, whereby participants would place their arm through a plastic bag with a whole in to minimize air escaping, before the small port in the door was opened. Activity was recorded with a Doppler radar.

We measured overall 24‐h energy expenditure, including overnight resting metabolic rate (RMR: from midnight to 6 AM), post‐prandial energy expenditure (PPEE: between 1:30 PM and 3:30 PM) and exercise‐induced energy expenditure (EIEE: in response to a standard stepping exercise from 4:30 PM to 4:45 PM).

### 2.6 24‐h HMRU metabolic study

**FIGURE 1:**
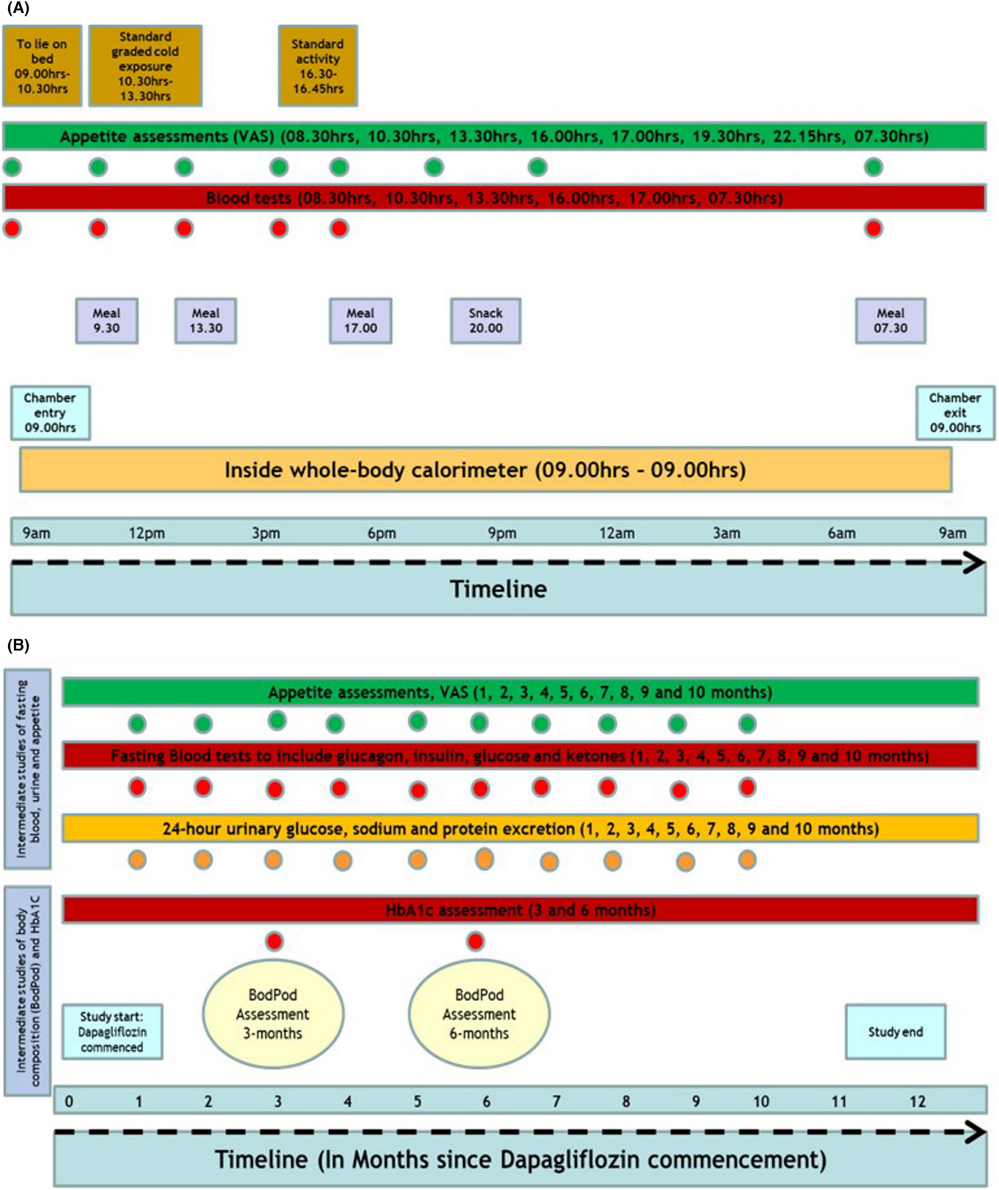
A schematic diagram of the study design. (A) 24‐h HMRU study protocol (baseline and at 12‐months). (B) Timeline of intermediate studies between baseline and 12‐months

Participants entered the metabolic chamber at 9 AM, with breakfast at 9:30 AM and lunch at 1:30 PM. At 4:30 PM, there was a standard activity protocol involving stepping for 15 min at a rate of one full‐step up and down per second. This enabled assessment of EIEE. Participants were provided with a standard meal and snack at 5:00 PM and 8:00 PM, respectively. Basal metabolic rate [BMR] was calculated through use of the ‘Katch‐Mcardle formula’: BMR = 370 + [21.6 × Lean Body Mass (kg)].^13^ RMR was calculated from BMR through use of the following formula: RMR = BMR × 1.25. This was used for adjusting meal portions to match individual caloric requirements. Participants were asked to sleep from 10:30 PM till 7 AM the next day. Breakfast was provided at 7:30 AM with chamber exit at 09.00 AM. A schematic diagram of the 24‐h HMRU study design is summarized in Figure 1.

### 2.7 SGLT2 inhibitor (Dapagliflozin) treatment

Following the baseline HMRU metabolic study, each participant commenced Dapagliflozin (10 mg, orally, once per day). All participants took Dapagliflozin therapy for the entire 12‐month duration of the study (following their initial baseline HMRU study).

### 2.8 Appetite scores

Participants had monthly assessments of appetite measures (standard visual analogue score [VAS]) and fasting blood tests. Analytes included fasting leptin and adiponectin (assays detailed below). The VAS applied is used routinely for the assessment of appetite and has been validated previously.^14^

### 2.9 Dietary intervention

Each participant received one‐to‐one dietary input provided by specialist weight management dieticians for the entire duration of the study. Specifically, participants were advised on a low carbohydrate diet (<100 g/day), with predominantly protein and fat (mainly from mono and polyunsaturated fats), and 400 g (5 portions) of vegetables/day. Records of food intake (including the estimated daily carbohydrate intake as <100 g, 100–300 g and >300 g) were encouraged and reviewed at each of the monthly assessments during a week.

### 2.10 Biochemical assays

Fasting blood samples were collected into EDTA and serum separating tubes (SST) for plasma and serum analyses, respectively. Samples from baseline and months 1, 2, 3, 6 and 12 were analysed for leptin and adiponectin. For both leptin and adiponectin measures, we used a sandwich enzyme immunoassay, with an assay range of 15.6–1000 pg/ml and 3.9–250 ng/ml for leptin and adiponectin, respectively, (ranges for diluted sample). Both assays were supplied by R&D systems.

### 2.11 Statistical analysis

Based on existing randomized controlled studies and real‐world data for Dapagliflozin,^15^ we estimated a mean weight loss of around 4 kg at 12 months following initiation onto Dapagliflozin therapy. On this basis, assuming a standard deviation of 5 kg, a priori power calculations suggested a sample size of 18 participants would provide 80% power, at the 5% level, to detect a change in body weight of 4 kg. As good data for body weight loss were available prior to study commencement and this was expected to have a relatively large variance, the study was powered to detect significant changes in this variable. As such, the study was also expected to have high power to detect significant changes in energy expenditure, body composition and appetite following 12‐months therapy with Dapagliflozin.

Data collected at multiple pre‐defined time points enabled assessment of temporal changes in the continuous variables. Graphical data displays guided the choice of statistical model used for data analysis. Quantile‐quantile (Q‐Q) plots were used to test the assumption that all data were approximately normally distributed. Data from hormonal parameters were right‐skewed and were therefore log‐transformed prior to analysis.

For analysis where only two time points were compared (e.g. baseline and month 12 for weight, body composition and energy expenditure), a paired Student t‐test was used for testing. For analysis of multiple time points (e.g. monthly changes in body weight), a ‘linear mixed‐effects (repeated measures) regression model’ was used to quantify and derive inferences from the data, as it accounts for the correlation between repeated observations made on participants.^16^ A mixed model enables more efficient estimation of the fixed effects (time) and yields more robust statistical tests than ANOVA.^17^ For data where log transformations were used, a mixed‐effect model was run on both non‐transformed and log‐transformed data. All analyses reported were based on complete cases, and inferences made under the assumption that data were missing at random. *p*‐values <.05 were considered statistically significant. SPSS version 27 was used for all analyses. Graphs were created using all available data in Excel.

## 3 RESULTS

### 3.1 Baseline participant data

Seventeen of the recruited participants (n=18) completed the entire study (one participant missed the final metabolic study but attended all other appointments). The participants included 7 women and 11 men, baseline mean age 51 years (SD 9.2), mean body weight 129.4 kg (SD 25.9, range 90–190.8 kg) and mean BMI 46.1 kg/m^2^ (SD 8.3). Mean duration of T2D at recruitment was 4.8 years (SD 3.2). Two participants reported their identity as black British, three as Asian, one as mixed ethnicity, and twelve as white British. All patients were on metformin monotherapy for glycaemic control at recruitment, and six were also on statin therapy. Baseline and 12 months follow‐up metabolic parameters are included in Table S1.

### 3.2 Dietary changes

Given that the majority of participants did not record accurate estimates of carbohydrate (CHO) intake, and the relatively small number of participants overall, to have reported these data would not have been clinically meaningful. Therefore, we took the decision not to include such data. The lack of estimate of dietary carbohydrate content was due to multiple reasons including impaired self‐recall and inadequate compliance.

### 3.3 Changes in energy expenditure

Full 24‐h assessment of energy expenditure (between baseline and 12 months follow‐up and ba) is reported for 14 participants; 3 patients did not want to stay overnight in the metabolic chamber for their final metabolic assessment (although all other data were collected).

There was no change in any measures of energy expenditure between baseline and 12‐month assessments (including total energy expenditure, PPEE, REE and EIEE: data shown in Table 2). There was also no change in energy expenditure per kg of lean mass between baseline and month 12 (*p* = .203).

**TABLE 2:** Changes in energy expenditure, body composition, adiponectin and leptin (analysed with paired t‐test)

Measure	Baseline Mean (SD)	12‐month Follow‐up Mean (SD)	*p* value
Domains of energy expenditure			
Total measured 24‐h energy expenditure (kcal), *n* = 14	2685 (574)	2559 (466)	.055
Total estimated 24‐h energy expenditure (kcal), *n* = 14	2134 (289)	2130 (367)	.954
Post prandial energy expenditure (kcal), *n* = 14	310 (49)	299 (52)	.147
Overnight resting energy expenditure (kcal), *n* = 14	616 (108)	603 (490)	.384
Exercise‐induced energy expenditure (kcal), *n* = 14	265 (69)	254 (52)	.172
Body composition			
Weight (kg), *n* = 17	131.1 (25.7)	123 (24.2)	.009
Muscle mass (kg), *n* = 16	62.5 (10.6)	63.6 (10.5)	.306
Fat mass (kg), *n* = 16	71.1 (23.6)	61.2 (23.3)	.002
Biochemistry			
Adiponectin (ng/ml), *n* = 17	5898 (4329)	6099 (3653)	.699
Leptin (pg/ml), *n* = 17	54,135 (31,260)	42,880 (26,005)	.011

### 3.4 Body weight and body composition changes

**FIGURE 2:**
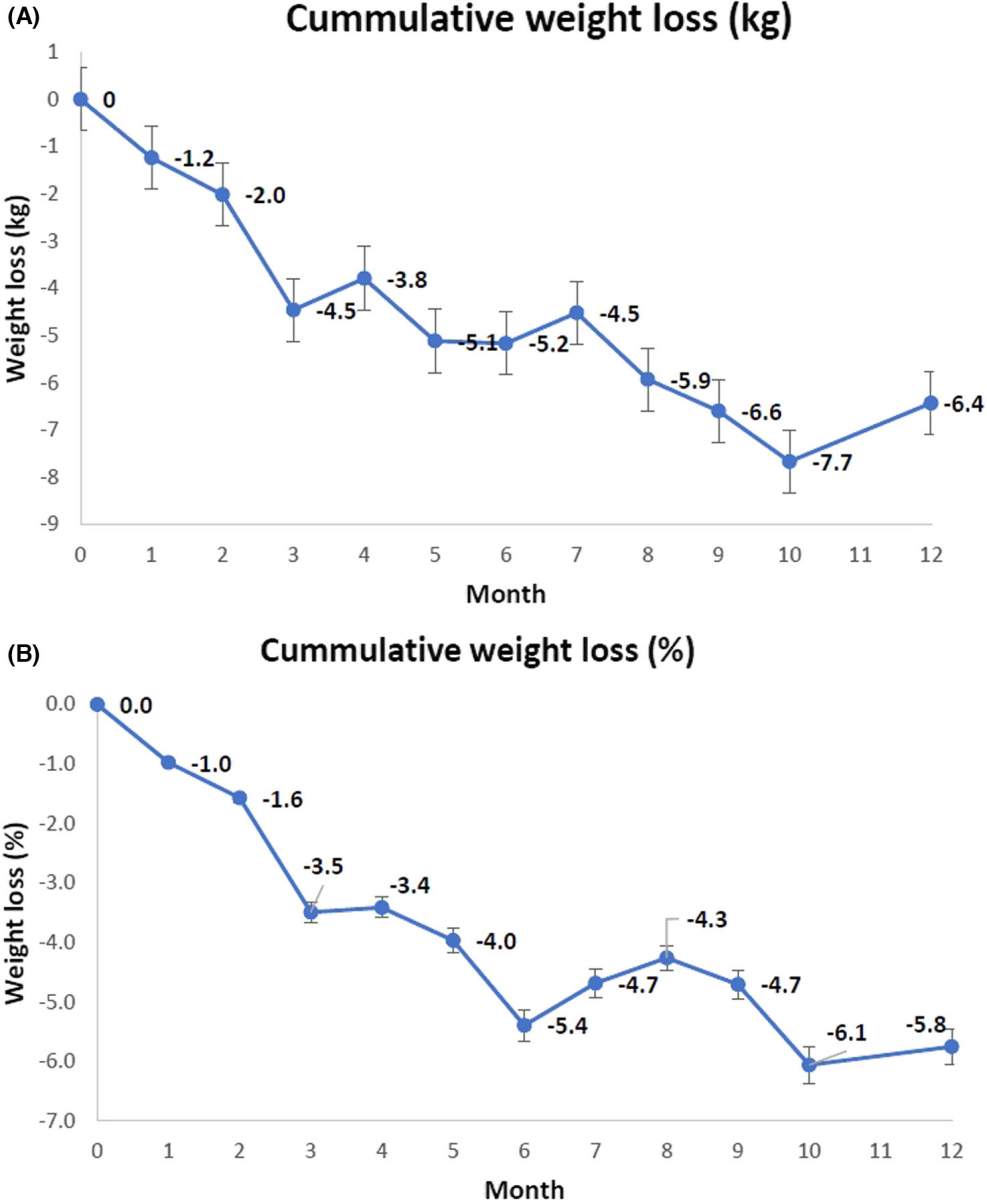
Cumulative weight loss, with bars representing standard error of the mean. (A) Cumulative weight loss (kg), (B) Cumulative weight loss (%)

*Temporal changes in body weight:* Mean body weights at baseline, 6 and 12 months were as follows: 129.4 kg (SD 25.9 kg), 124.2 kg (SD 26.2 kg) and 123 kg (SD 24.2 kg), respectively. Figure 2 shows cumulative changes to body weight and percentage weight loss based on all available data. Individual changes in body weight are included in Table S2.

A paired Student *t*‐test was used to analyse the difference between baseline and 12‐month follow‐up data, for the 17 participants where data were available (data shown in Table 2, weight loss of 8.1 kg, paired t‐test *p* value .009). Fitting a mixed‐effect model gave an estimate for the intercept (starting weight) of 129.1 kg, (SEM of 6 kg) and a monthly change in body weight of −0.72 kg (SEM 0.1 kg), resulting in a calculated mean body weight of 120.7 kg at 12 months follow‐up. Therefore, using this model, compared with baseline data there was a total predicted loss of body weight of 8.6 kg at 12 months (likelihood ratio test *p* < .001) in response to Dapagliflozin therapy combined with dietary carbohydrate restriction.

*Temporal changes in body composition:* Comparison with baseline data for body composition was based on 16 participants for the 12‐month data. The mean muscle mass for baseline and 12‐month follow‐up assessments was 62.5 kg (10.6) and 63.6 kg (10.5), respectively (paired t‐test *p* = .306). Similarly, there was no significant difference in the monthly rate of change in muscle mass as calculated by the mixed‐effect model (gradient of change 0.03 kg, likelihood ratio test *p* = .762). Data are shown in Table 2. Individual changes in lean and fat mass are included in Figures S1 and S2.

Mean fat mass at baseline and at 12 months follow‐up assessments was 71.1 kg (SD 23.6) and 61.2 kg (23.3), respectively (paired t‐test *p* = .002, data shown in Table 2). A mixed‐effect model revealed that monthly reduction in fat mas was statistically significant (likelihood ratio test *p* < .001), with a monthly gradient of −0.74 kg (SEM 0.2 kg). Based on the mixed‐effect model, the calculated loss of fat mass at 12 months follow‐up was 8.9 kg. The reason for the fat loss exceeding overall weight loss was the numerical (albeit non‐significant) increase in lean mass following Dapagliflozin administration. There was a statistically significant Pearson correlation of 0.311 (*p* = .028) between loss of body weight and fat mass.

### 3.5 Changes in appetite

**FIGURE 3:**
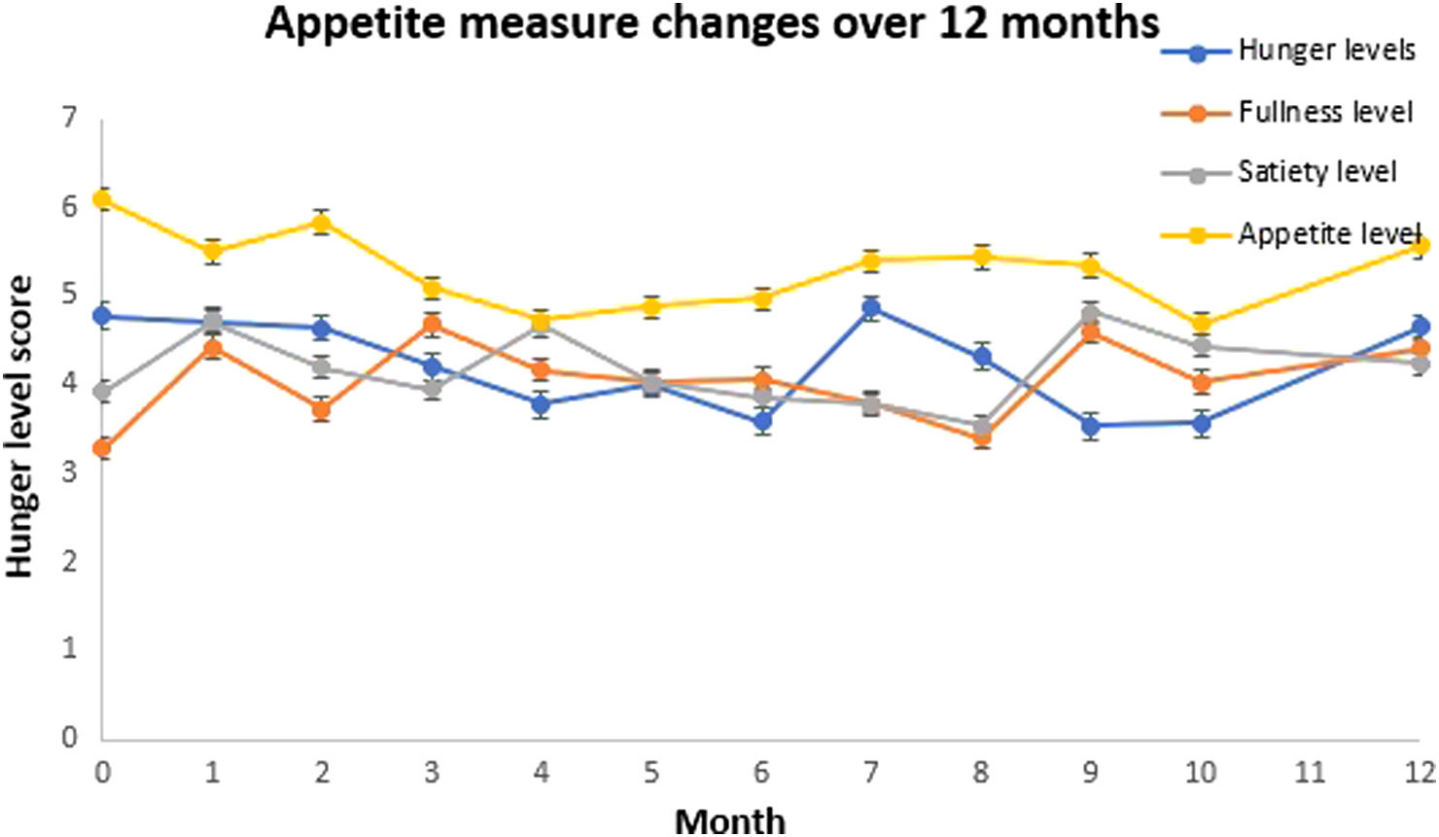
Temporal changes in appetite measures, with bars representing standard error of the mean

Among all participants, there were 198 completed assessments of appetite. Compared with baseline data, there were no statistically significant differences in self‐reported measures of appetite (including fasting hunger, satiety and fullness scores) at any of the follow‐up assessments over 12 months (Figure 3).

### 3.6 Leptin and adiponectin

**FIGURE 4:**
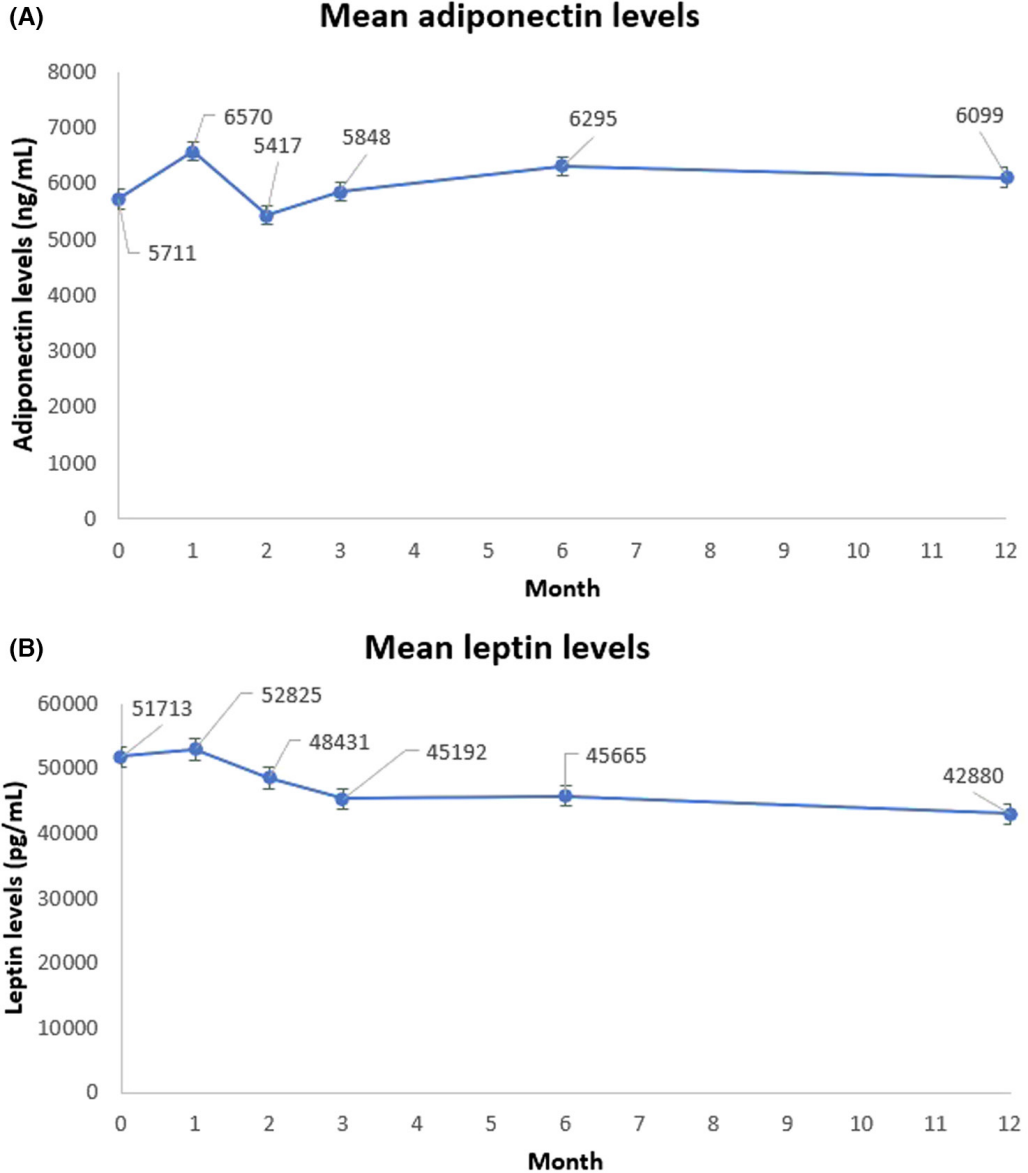
(A) Mean adiponectin levels over time (bars represent standard errors). (B) Mean leptin levels over time (bars represent standard errors)

Mean fasting serum adiponectin level at baseline (data available for 18 participants) and 12 months (data available for 17 participants) was 5711 ng/ml (SD 4275) and 6099 ng/ml (SD 3653), respectively (Figure 4).

There was no difference between baseline and month 12 adiponectin levels (*p* = .699 using paired Student *t*‐test, Table 2). Similarly, there was no significant monthly change in serum adiponectin levels based on a mixed‐effect model (likelihood ratio test *p* = .761).

Mean serum leptin level at baseline (data available for 18 participants) and 12 months (data available from 17 participants) was 51,713 pg/ml (SD 32,021) and 42,880 pg/ml (SD 26,005), respectively (Figure 4). There was a significant reduction of 11,255 pg/ml at month 12 (*p* = .011 using paired Student *t*‐test, Table 2).

Similarly, there was a statistically significant monthly gradient of −852 pg/ml (likelihood ratio test *p* < .001) based on a mixed‐effect model. This would lead to a calculated level of leptin of 40,258 pg/ml at 12 months follow‐up.

Statistical analyses from log‐transformed data provided similar results and inferences as the paired Student *t*‐test and mixed‐effect model for both adiponectin and leptin. Pearson correlation analysis showed a significant correlation between changes in body fat mass and serum leptin levels over the 12‐month period of follow‐up (correlation coefficient 0.978, *p* = .02).

## 4 DISCUSSION

We provide detailed characterization of metabolic and appetitive responses to 12 months of Dapagliflozin therapy in combination with dietary carbohydrate restriction in patients with T2D and class 3 obesity. We demonstrate significant and substantial reduction in body weight and fat mass, with significant reduction in serum levels of leptin, despite preservation of 24‐h energy expenditure and self‐reported appetite. To our knowledge, our study is the first to report a comprehensive 24‐h metabolic assessment in response to SGLT2i therapy, beyond 12 weeks. Our observation of maintenance of constant metabolic rate with SGLT2i therapy (despite weight loss) reflects previous reports in the literature.^18^ Explanations may include preserved muscle mass, and possible direct effects of Dapagliflozin on thecontrol of energy expenditure.

We demonstrated a mean body fat mass loss of 9.9 kg with Dapagliflozin over 12 months. However, the study size and design limits generalisability of our findings. One explanation is that we combined Dapagliflozin with a low carbohydrate diet (LCD). Participants were encouraged to restrict their dietary intake of carbohydrates to 100 g per day, although none managed to adhere strictly to a LCD of this extent. However, each participant received dietary education and changed their diets accordingly. Thomas and Cherney^19^ suggested that increased dietary adherence may help to optimize weight loss in response to the administration of SGLT2i therapies. We hypothesize that focus on dietary carbohydrate restriction helped to mitigate against SGLT2i‐induced propensity for increased dietary carbohydrate intake, with important implications for broader implementation of combination SGLT2i/dietary carbohydrate restriction in routine clinical practice. However, a small but real risk of euglycaemic diabetic ketoacidosis (euDKA) needs to be considered in patients on SGLT2i. EuDKA can affect patients with both Type 1 Diabetes (T1D) and T2D, and is characterized by the presence of ketones, acidosis and normal blood sugar level. It is a rare side effect of treatment with SGLT2i therapies, and was first described in patient with T2DM and Prader‐Willi syndrome on a LCD by Hayami et al.^20^ and in two patients with T2D following elective surgery.^21^ As it is a potentially dangerous side effect of SGLT2i therapies, prompt testing for urinary or plasma ketones, with plasma pH is important in patients who present with malaise, abdominal pain, vomiting or respiratory distress. The risk of developing euDKA is much higher in patients with T1D, and therefore it is important to establish the correct subtype of Diabetes Mellitus for a patient who is started on a SGLT2i therapy.^21^ A risk assessment should be performed by a diabetes clinician prior to the recommendation of a LCD concomitant with the administration of a SGLT2i therapy in T2D.

Existing evidence for SGLT2i therapies suggests either appetite enhancement^22^ or no effect on appetite.^23^ Among participants with T2D, Dapagliflozin (5 mg) was shown to increase appetite (at 3 months) for sugar‐rich foods.^24^ Absence of SGLT2i‐induced appetite enhancement in our study is surprising as serum levels of appetite hormones often change following weight loss.^25^ The LCD itself may have mitigated against SGLT2i‐induced appetite enhancement, through reduced competition of insulin with leptin within hypothalamic appetite suppression pathways. Reductions in fat mass may have improved insulin and leptin resistance, thereby optimizing the appetite suppressant effects of leptin. Furthermore, despite VAS being used in studies with GLP1 analogues,^26^ such self‐reported hunger scores may not accurately reflect appetite.

A recent meta‐analysis (with 3–6 months follow‐up) showed that therapy with SGLT2i therapies associate with a significant reduction in serum leptin by 290 pg/ml.^27^ We demonstrated a much greater reduction in serum leptin in response to Dapagliflozin therapy (852 pg/mL per month). One explanation for this discrepancy is the greater loss of body fat mass in our study compared with that in other studies. Our data on serum adiponectin changes are less easily explained. Imbeault et al.^28^ showed that serum adiponectin concentration increased following 8–9% weight loss. This was further supported by data reported by Lin et al.^29^ with serum levels of adiponectin negatively correlating with changes in body fat mass. However, other studies have not shown any rise in serum (total) adiponectin levels following body weight loss, possibly due to a redistribution of adiponectin oligomers in response to weight loss, not apparent when total adiponectin level is measured.^30^ Higher molecular weight adiponectin is a better predictor of glucose itolerance than total adiponectin.^31^ In our study, it is possible that the reduction of body fat mass was insufficient in magnitude or duration for changes in serum (total) adiponectin to occur.

The main limitation of our study was a relatively small sample size. This was entirely pragmatic, reflecting the complexity of study assessments and cost. Although one participant dropped out at month 12, we observed significant effects of Dapagliflozin, indicating that the study was still sufficiently powered. As participants did not fully adhere to strict dietary carbohydrate restriction, it was difficult to draw accurate conclusions regarding the impact of dietary carbohydrate restriction on the observed results. As this was a mechanistic study, there was no control group.

To conclude, this is one of the most comprehensively‐phenotyped studies of the metabolic and appetitive effects of an SGLT2i therapy in T2D reported on to date. The combination of Dapagliflozin therapy with dietary carbohydrate restriction for 12 months in patients with T2D and class 3 obesity resulted in a significant and substantial reduction of body weight, fat mass and serum leptin, but without any discernible compensatory changes in metabolic rate or appetite. Our study promotes a need for a randomized controlled trial investigating the metabolic effects of SGLT2i therapies combined with LCD in people withT2D and obesity.

## AUTHOR CONTRIBUTIONS

**Petra Hanson:** Data curation (equal); formal analysis (equal); investigation (equal); project administration (equal); writing – original draft (equal); writing – review and editing (equal). **Harpal Randeva:** Conceptualization (equal); supervision (equal); writing – review and editing (equal). **Daniel J Cutbertson:** Conceptualization (equal); writing – review and editing (equal). **Paul O'Hare:** Conceptualization (equal); supervision (equal); writing – review and editing (equal). **Nick Parsons:** Conceptualization (equal); data curation (equal); formal analysis (equal); methodology (equal); writing – review and editing (equal). **Kamaljit Chatha:** Formal analysis (equal); investigation (equal); resources (equal); writing – review and editing (equal). **Gemma Reidy:** Formal analysis (equal); investigation (equal); methodology (equal); resources (equal); writing – review and editing (equal). **Martin O Weickert:** Conceptualization (equal); writing – review and editing (equal). **Thomas M Barber:** Conceptualization (equal); funding acquisition (equal); methodology (equal); resources (equal); supervision (equal); writing – original draft (equal); writing – review and editing (equal).

## FUNDING INFORMATION

Grants supporting this project: Investigator‐initiated research study funded by AstraZeneca (Thomas M Barber was PI on this grant). Role of study funder: The study funder was not involved in the design of the study; the collection, analysis and interpretation of data; writing the report; and did not impose any restrictions regarding the publication of the report.

## CONFLICT OF INTEREST

None of the authors has any conflict of interest. This study was funded by AstraZeneca.

## Supporting information


Figure S1

Figure S2

Table S1

Table S2

## Data Availability

Data are available on request from the corresponding author.
