# Supplementary figures and images for: The DAPA‐DIET study: Metabolic response to Dapagliflozin combined with dietary carbohydrate restriction in patients with Type 2 Diabetes Mellitus and Obesity—A longitudinal cohort study
Source: Endocrinol Diabetes Metab. 2022 Oct 20;5(6):e381. doi: 10.1002/edm2.381 (PMC9659664; doi:10.1002/edm2.381)

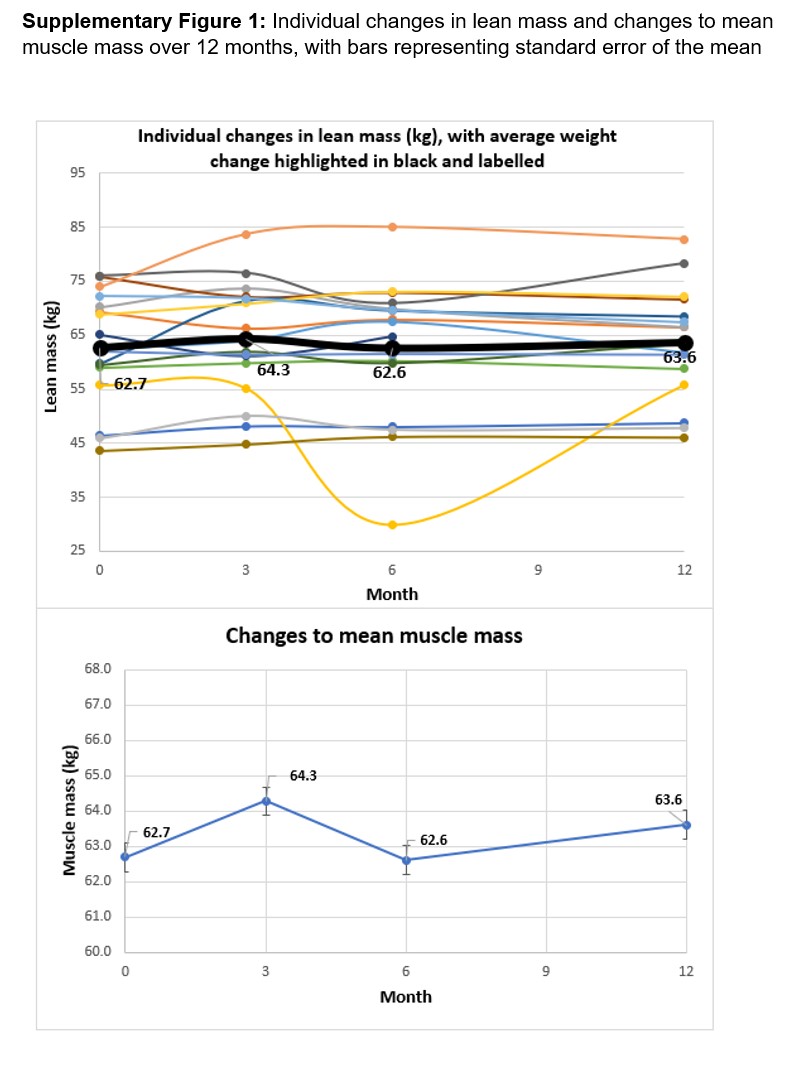

Supplement: Supplementary file 1 — Figure S1 [file EDM2-5-e381-s004.jpg]

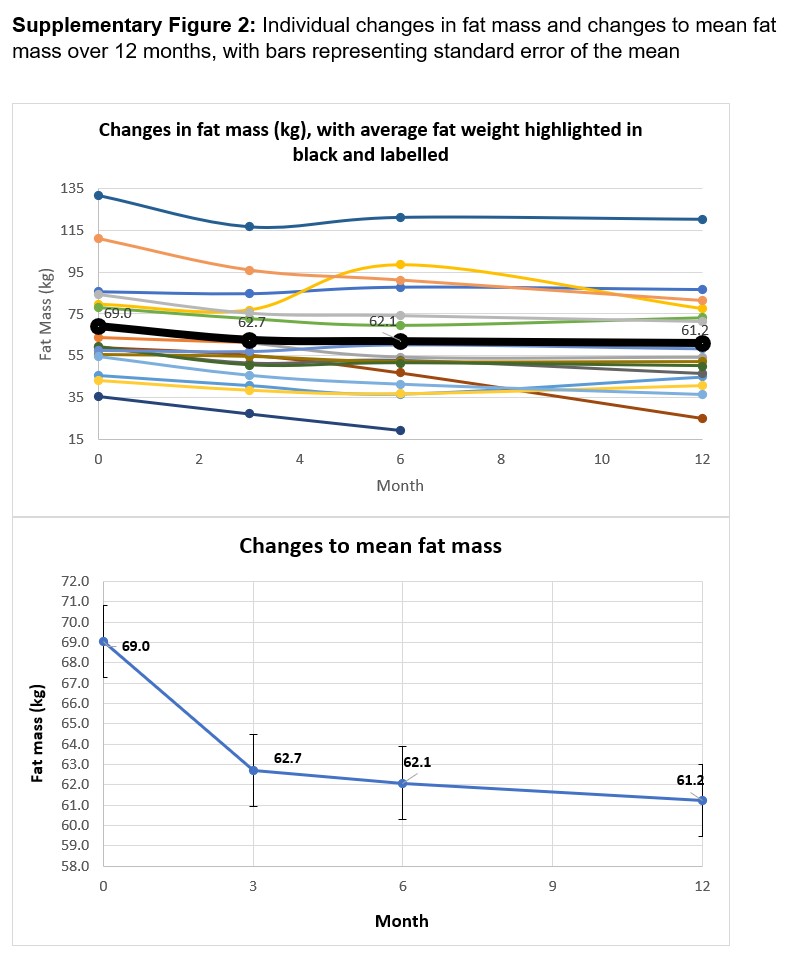

Supplement: Supplementary file 2 — Figure S2 [file EDM2-5-e381-s001.jpg]
