# Supplementary material for: The DAPA‐DIET study: Metabolic response to Dapagliflozin combined with dietary carbohydrate restriction in patients with Type 2 Diabetes Mellitus and Obesity—A longitudinal cohort study
Source: Endocrinol Diabetes Metab. 2022 Oct 20;5(6):e381. doi: 10.1002/edm2.381 (PMC9659664; doi:10.1002/edm2.381)
Supplement: Supplementary file 3 — Table S1 [file EDM2-5-e381-s003.docx]

**Supplementary material**

**Supplementary Table 1:** Paired student t-test comparing metabolic parameters

| **Metabolic parameter** | **Mean baseline (SD)** | **Mean one year**  **(SD)** | **The difference in means between one year and baseline (SD)** | **Lower 95% CI** | **Upper 95% CI** | **Sample size** | **P value** |
| --- | --- | --- | --- | --- | --- | --- | --- |
| **HbA1c (mmol/mol)** | 53.9 (14.1) | 52.7 (19.0) | -1.3 (9.2) | -6.0 | 3.4 | 17 | 0.569 |
| **Total cholesterol (mmol/L)** | 4.4 (1.1) | 4.3 (1.2) | -0.1 (0.7) | -0.5 | 0.2 | 17 | 0.52 |
| **Triglycerides (mmol/L)** | 2.8 (2.7) | 2.1 (1.3) | -0.7 (1.8) | -1.6 | 0.2 | 17 | 0.132 |
| **Alt (IU/L)** | 28.6 (14.0) | 28.8 (19.4) | 0.3 (9.8) | 5 | -5.5 | 16 | 0.92 |
| **TSH (mU/L)** | 1.9 (0.8) | 1.9 (0.8) | 0 (0.50 | 0.3 | -0.3 | 15 | 0.943 |
| **Creatinine (µmol/L)** | 72.6 (16.7) | 70.4 (15.5) | -2.2 (9.9) | -7.1 | 2.8 | 18 | 0.368 |
| **Urea (mmol/L)** | 5.5 (2.1) | 5.4 (1.3) | 0 (1.9) | -1.0 | 0.9 | 18 | 0.921 |

**Supplementary Table 2:** Complete data set for available weights (kg)

| **Participant** | **Baseline** | **Month 1** | **Month 2** | **Month 3** | **Month 4** | **Month 5** | **Month 6** | **Month 7** | **Month 8** | **Month 9** | **Month 10** | **Month 12** |
| --- | --- | --- | --- | --- | --- | --- | --- | --- | --- | --- | --- | --- |
| 1 | 132 | 132.8 | 131.2 | 132.7 | 132.6 | 132.4 | 135.8 | 134.4 | 133.6 | 132.6 | 134.6 | 135.4 |
| 2 | 133.2 | 132.6 | 130.2 | 127.6 | 129 | 128.6 | 128.7 | 130.6 | 130.4 | 132.6 | 128.4 | 127.4 |
| 3 | 139.6 | 138.4 | 140.2 | 134.7 | 131.4 | 129.4 | 124.2 | 124 | 123.8 | 123.4 |  | 120.6 |
| 4 | 135.4 | 133.8 | 134.4 | 131.9 | 132.8 | 132.6 | 128 | 126.5 | 129.6 | 130.7 | 131.6 | 132.4 |
| 5 | 108.1 | 105.6 | 105 | 104.7 | 104.8 | 105.4 | 104.1 | 104.8 | 104.4 | 104 | 106.4 | 106.7 |
| 6 | 137 | 136 | 134.2 | 132.7 | 131.3 | 132 | 130 | 131 | 132 | 132.2 | 131.6 | 131.9 |
| 7 | 100.8 | 96 | 94.2 | 88.4 | 88.8 | 88.8 | 84.2 | 83.5 | 84.8 | 83.8 | 83 |  |
| 8 | 134.9 | 135.2 | 132.4 | 127.4 | 127.2 | 119.6 | 119.7 |  |  |  | 100 | 96.7 |
| 9 | 135 | 134.6 | 134.4 | 127.9 | 126.8 | 127.2 | 123.6 | 127.2 | 126.4 | 126.4 | 124.8 | 124.6 |
| 10 | 99 | 99.2 | 101.8 | 99.3 | 99.8 | 99 |  |  | 98.3 | 98 | 99.2 | 98.2 |
| 11 | 190.8 | 187.8 | 187.4 | 188.1 | 191 | 190.2 | 190.9 | 189.6 | 190.2 | 188 | 187 | 188.9 |
| 12 | 118.9 | 118.6 | 116.8 | 112.4 |  | 112.8 | 111 | 111.4 | 111.6 | 113.2 | 113.6 | 113.5 |
| 13 | 119.5 | 119.2 | 117.6 | 118.5 | 117.4 | 119.2 | 121.5 | 121.4 | 122.4 | 120.6 | 120.2 | 119.7 |
| 14 | 185.2 | 182.2 | 183.8 | 179.6 | 179.6 | 180.4 | 176.1 | 176.8 | 173 | 167.4 | 167.8 | 164.1 |
| 15 | 90 | 88 | 90.2 | 90.4 | 91.6 | 91.4 | 91.2 | 92.2 | 93.2 | 92.6 | 93 | 94 |
| 16 | 130.4 | 128.4 | 126.6 | 125.5 | 123.6 | 122 | 121.8 | 120 | 121.8 | 121.4 | 121.8 | 119.3 |
| 17 | 112.3 | 113.4 | 110.8 | 109.5 | 111 | 111.4 | 110 | 114.4 | 114.6 | 114.8 |  | 113 |
| 18 | 127 | 125 | 121.6 | 117.5 | 116.6 | 114.6 | 111 | 110.2 | 108.8 | 105.8 | 104.6 | 103.9 |
